# Effective Gold Biosorption by Electrospun and Electrosprayed Bio-Composites with Immobilized *Lysinibacillus sphaericus* CBAM5

**DOI:** 10.3390/nano10030408

**Published:** 2020-02-26

**Authors:** Carolina Páez-Vélez, J. L. Castro-Mayorga, Jenny Dussán

**Affiliations:** 1 Microbiological Research Center (CIMIC), Department of Biological Sciences, Universidad de Los Andes, Bogotá 111711, Colombia; c.paez1660@uniandes.edu.co; 2 Nanobiotechnology and applied microbiology (NANOBIOT), Department of Biological Sciences, Universidad de Los Andes, Bogotá 111711, Colombia; jl.castrom@uniandes.edu.co

**Keywords:** gold, electrospinning, electrospraying, polycaprolactone, alginate, *Lysinibacillus sphaericus* CBAM5

## Abstract

Electro-hydrodynamic processing, comprising electrospinning and electrospraying techniques, is a novel technology used in the production of nano- and sub-micro-scale materials with specific properties suitable for environmental remediation processes. Polycaprolactone (PCL) micro-fibrous mats and alginate microcapsules were produced using electrospinning and electrospraying techniques respectively, and *Lysinibacillus sphaericus* CBAM5, a bacterium capable of metal removal by adsorption and accumulation inside the cell, was immobilized in these matrices. The polymeric structure was able to protect and maintain cell viability and the bio-composite materials were used to capture gold from synthetic water samples. The micro-fibrous membranes with immobilized bacteria were able to remove 93% of the gold after 120 h of inclusion in the aqueous medium. Using a filtration system, an efficiency of 64% was obtained for the removal of the precious metal after 10 cycles of filtration (2 h of exposure to the gold solution). In contrast, the microencapsulated *L. sphaericus* CBAM5 captured 64% of the gold after 4 h of the assay. Thus, both micro-structured matrices were suitable for the immobilization and protection of *L. sphaericus* CBAM5 and they showed high efficiencies of gold biosorption. Hence, these bio-composite materials could be used to concentrate gold from industrial wastewaters.

## 1. Introduction

One of the major challenges for humanity nowadays is to provide clean water for the entire global societies. Population growth, contamination of water sources by industrial activities, and droughts among other conditions, hamper efficient water supply in modern communities [1]. Mining and metallurgical operations are responsible for a great proportion of water contamination by metal ions, a type of pollutant that requires complex technologies for its removal from water bodies [2]. 

Some of the techniques used currently to remove metals include chemical precipitation, sorption, coagulation, and oxidation/reduction, among others [2]. Sorption seems to work as a good alternative in the capture of metal ions due to its low cost, simple operation, its lack of byproducts, and the regeneration of the starting materials [2]. Sorption also makes it possible to recover and recycle metal ions from wastewaters with interesting properties for industrial applications. Gold is a clear example of this. This precious metal is characterized by its slow corrosion, high conductivity, and its nanoparticles are currently being implemented in the electronic, biomedical, and pharmaceutical industries [3].

The past two decades have seen rapid advances in the field of nanotechnology and several new applications have emerged from these studies. The obtained nanomaterials are characterized for having large surface areas, stability, adequate mechanical properties, and low toxicity, among others [2]. Due to these specific properties, several nanomaterials have been used in environmental sciences as an alternative in remediation processes [1]. Particularly in the field of water treatment, the removal of pollutants is a great challenge and a current problem due to increased industrial activity, such as mining and metallurgy [2]. There has therefore been an increment in the use of nanostructured components because they have shown several advantages over traditional water treatment techniques as a result of their large surface areas and reactivity [1].

In recent years, a top-down technique called electro-hydrodynamic processing (EHP) has been used to manufacture nanomaterials. It consists in the injection of a polymer solution exposed to a high voltage which creates a fine, elongated jet that determines the diameter of the electrospun fibers or electrosprayed capsules formed [4]. This is a simple and effective technique with applications at the industrial level, given that it can be scaled-up [5].

In the case of water treatment, adsorption of pollutants has shown considerable advantages, thus several nanomaterials have been used for such applications [6]. Nano-adsorbent materials from organic polymers obtained by EHP are known to have large surface areas and high porosity that allow the capture of heavy metals from aqueous media [5]. Such nano-adsorbents can be obtained from polymers like polycaprolactone (PCL) and sodium alginate and they are biodegradable, easy to manipulate, and biocompatible, among others [7,8]. These polymers have also been used as immobilization matrices for bacteria with metabolic potential for remediation processes in the environment. These types of bio-composite materials have been studied for the removal of textile dyes, heavy metals, and other water pollutants [9,10,11].

*Lysinibacillus sphaericus* is a Gram-positive bacterium that has been extensively studied for its ability to resist and capture metals such as uranium, Pb(II), Cr(IV), Cd(II), Hg(II), and Au(III) [12,13,14,15,16]. It is known to have a surface layer (S-layer) protein, which is a porous lattice that protects it and acts as an ion trap. The strain *L. sphaericus* CBAM5 has shown a great potential to capture several metal ions like gold and mercury [15,16,17]. A study of its genomic sequence has shown that it possesses several coding regions for S-Layer proteins and it presents heavy metal efflux pumps that help the cell to resist high concentrations of metal ions [13,18]. 

Accordingly, the aim of this study was to synthesize and characterize micro-fibrous membranes of PCL and microcapsules of alginate. The immobilization of living cells of *L. sphaericus* CBAM5 in these two types of materials was performed. Additionally, the gold biosorption efficiency of these bio-composite materials was determined by using different methods of exposure to water samples containing the metal ion. 

## 2. Materials and Methods 

### 2.1. Bacterial Growth and Media

The selected strain, *L. sphaericus* CBAM5, was incubated in nutrient agar (OXOID) for 24 h at 30 °C. The biomass was then collected and resuspended in minimum salt medium (MSM). This medium contains: KH_2_PO_4_ 0.5 g/L, NH_4_Cl 1 g/L, Na_2_SO_4_ 2 g/L, KNO_3_ 2 g/L, CaCl_2_ 0.001 g/L, FeSO_4_ 0.0004 g/L, MgSO_4_·7H_2_O 1 g/L, and sodium acetate 5 g/L. The resuspended bacteria had a concentration of approximately 10^8^–10^9^ colony-forming units (CFU)/mL and was then used for the microencapsulation and immobilization assays.

### 2.2. Production of Bio-Composite Microstructures

**Figure 1:**
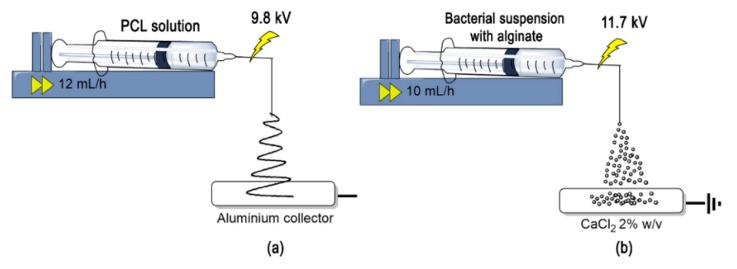
Representation of the electrohydrodynamic processing used to obtain (**a**) micro-fibrous mats of Polycaprolactone (PCL) and (**b**) microcapsules of alginate with *L. sphaericus* CBAM5.

All the sub-micro-structured materials were synthesized using an EHP machine (NANOFIB1000) with a flow pump BYZ-810D, as shown in Figure 1, manufactured and commercialized by Qubitextp (Bogotá, Colombia).

#### 2.2.1. Electrospinning of Polycaprolactone (PCL) Micro-Fibrous Mats

The PCL solution was prepared by dissolving 8% (wt./wt.) of polymer in a chloroform/1-butanol 75:25 (wt/wt) mixture, both reagent grades, at room temperature and with magnetic stirring. The PCL solution was electrospun for 3 h using an EHP machine (Figure 1a). The solutions were then processed at room temperature under a constant flow of 10 mL/h using an 18-gauge injector, scanning vertically onto the metallic cylindrical collector covered with aluminum foil with an oscillatory movement at 1600 rpm. A dual polarization added voltage of 9.8 kV per single emitter and a tip-to-collector distance of 12 cm were used. 

The PCL micro-fibrous mat obtained was dried for 24 h in a desiccator and sterilized under UV light for 40 min on each side. 

#### 2.2.2. Immobilization by Adhesion of *L. sphaericus* CBAM5 in PCL Micro-Fibrous Mats

Six pieces of 5.5 × 6 cm and average weight of 0.13 ± 0.03 g of PCL membranes were placed in 50 mL flasks with 30 mL of a *L. sphaericus* CBAM5 suspension with a concentration of 1.02 × 10^9^ CFU/mL in saline solution (0.85%). The flasks were incubated at 30 °C, 150 rpm for 5 h. The membranes were then washed twice with sterile saline solution (0.85%) to remove non-immobilized cells. 

Bacterial immobilization was confirmed by scanning electron microscopy (SEM) analysis and a detachment protocol was used to estimate the bacterial concentration immobilized in the membranes. 

The detachment protocol was made as previously reported [11]. Firstly, three membranes were transferred to flasks containing 15 mL of sterile saline solution (0.85%) and these were vortexed for 30 s. Then, sonication was performed at 40 kHz and 4 °C in a Branson 2800 Ultrasonic cleaner during 2 cycles of 1 min sonication and 30 s rest for a total of 10 min of sonication. After sonication, the membranes were vortexed for 1 min and between each cycle, fresh saline solution (0.85%) was used. Standard serial 10-fold dilutions were inoculated (10 µL) in nutrient agar and incubated for 24 h at 30 °C for the drop plate count method [19].

#### 2.2.3. Microencapsulation of *L. sphaericus* CBAM5 in An Alginate Matrix by Wet Electrospraying

The bacteria suspension in MMS (90 mL) previously obtained (Section 2.1) was mixed with sodium alginate at 1%w/v. The solution was placed in a 20 mL syringe which was connected to a flow pump and was wet electrosprayed for 5 h into a CaCl_2_ 2%w/v solution in a collector covered with aluminum foil using an EHP machine (Figure 1b). The process was conducted at room temperature, under a constant flow of 10 mL/h using a 20-Gauge injector and scanning vertically onto the metallic collector. A dual polarization added voltage of 11.7 kV and a tip-to-collector distance of 20 cm were used. The bacteria-containing microparticles were filtrated, washed with distilled water, and stored at 4 °C until further use.

In order to quantify the bacterial concentration, 0.5 g of microparticles were crushed and mixed by placing them with 0.5 mL of sterile saline solution (0.85%) in a vortex. Standard serial 10-fold dilutions were inoculated (10 µL) in nutrient agar and incubated for 20 h at 30 °C for the drop plate count method [19].

### 2.3. Characterization of Electrospun and Electrosprayed Microstructures

#### 2.3.1. Scanning Electron Microscopy (SEM) and Energy Dispersive X-Ray Spectroscopy (EDS) Analysis

The micro-fibrous mats of PCL with and without immobilized bacteria were observed by scanning electron microscopy (SEM) using the JEOL JSM-6490LV (JEOL, Tokyo, Japan) scanning electron microscope equipped with an Oxford INCA PentaFetX3 energy dispersive X-ray spectroscopy (EDS) detector. Mats from the gold biosorption assays were also collected and sequentially washed with ethanol at 70%, 90%, and 100%. They were then placed in an aluminum support for metallization with graphite and SEM observation. A semi-quantification of gold with energy dispersive X-ray spectroscopy (EDS) was also performed. Estimation of the average fiber diameter was conducted by means of Image J software from 300 fibers at random from SEM images. 

#### 2.3.2. Optical Microscopy

The obtained microcapsules were observed using an optical microscope to measure the average diameter of the spheres. Several samples were placed in glass slides and observed in a light microscope Zeiss Axioskop 40 and different visual fields were photographed and analyzed by Image J to measure the diameter of the microcapsules. 

### 2.4. Gold Biosorption Assays with Electrospun and Electrosprayed Microstructures

#### 2.4.1. Biosorption by Inclusion of Micro-Fibrous Mats with Immobilized *L. sphaericus* CBAM5 in a Synthetic Water with Gold

For the biosorption assay, membranes with and without immobilized bacteria were placed in 30 mL of MSM spiked with HAuCl_4_ × 3H_2_O (10 mg/L). The mixture was incubated at 30 °C and 60 rpm. Aliquots of 1.5 mL were taken from the flasks at 0, 2, 4, 18, and 120 h. Flame atomic absorption spectrometry (FAAS) was used to analyze the gold concentration in the supernatants as described below. The bioassay was conducted in triplicate.

The sorption capacities (Ss) of the PCL sub-micro-fibrous mats with and without bacteria were calculated by Equation (1):

(1) $$Ss\left(\frac{mg}{g}\right)=\left(\frac{(C_{0}-C_{x})V}{W}\right),$$

where C_0_ is the initial gold concentration (mg/L) in the supernatant, C_x_ is the gold concentration in the aliquots taken at different times from the bioassay (mg/L), V is the volume used in the bioassay (L), and W is the total PCL mat weight (g). 

#### 2.4.2. Gold Biosorption Performance of Micro-Fibrous Mats with Immobilized *L. sphaericus* CBAM5 in a Filtration System

PCL mats with immobilized bacteria were wet with saline solution (0.85%) and then placed in a filtration system (MILLICUP™-FLEX, Millipore). Then, 30 mL of MMS spiked with 10 ppm of HAuCl_4_ × 3H_2_O were dripped over the filtration system with a constant flow of 150 mL/h (flow pump BYZ-810D) and gravity filtration was performed. The filtrate was collected, and 15 cycles of filtration were used. Aliquots of the filtrate were taken after 5, 10, and 15 cycles. Complete filtration time was 3 h. Finally, FAAS was used to analyze the gold concentration in the aliquots as described below. The bioassay was conducted in triplicate. The sorption capacities (S) of the PCL sub-micro-fibrous mats with and without bacteria were calculated by Equation (1).

#### 2.4.3. Biosorption of Gold by Microencapsulated Cells of *L. sphaericus* CBAM5 in Alginate

This assay was developed as previously reported [17]. Briefly, 5 g of alginate microcapsules with *L. sphaericus* CBAM5 were added to 15 mL of MSM spiked with HAuCl_4_ × 3H_2_O (60 mg/L) in 50 mL flasks. The mixture was incubated at 30 °C and 150 rpm. Aliquots of 1.5 mL were taken from the flasks at 0, 2, and 4 h. FAAS was used to analyze the gold concentration in the supernatants as described below. The bioassay was conducted in triplicate. 

The sorption capacities (S) of electrosprayed microcapsules with and without bacteria were calculated by Equation (2):

(2) $$S\left(\frac{mg}{g}\right)=\left(\frac{(C_{0}-C_{x})V}{W}\right),$$

where C_0_ is the initial gold concentration (mg/L) in the supernatant, C_x_ is the gold concentration in the aliquots taken at different times from the bioassay (mg/L), V is the volume used in the bioassay (L), and W is the total microcapsules wet weight (g). 

### 2.5. Flame Atomic Absorption Spectrometry (FAAS) Measurements

All the experiments conducted to quantify gold were carried out using a High-Resolution Continuum Source Atomic absorption spectrometer (HR-CSAAS, ContrAa 800, commercially available from Analytik Jena, Jena, Germany) in flame mode (FAAS). Flame conditions were properly optimized before taking the measurements, using the absorption line at 242.7590 nm. All gold standards were made on the day of measurement by diluting 1000 mg/L standard (HAuCl_4_, CertiPUR^®^, Merck, HCl 2 mole/L, Darmstadt, Germany) to concentrations between 0.5 and 10 ppm. Distilled water and hydrochloric acid 1 mol/L (ACS reagent, 37%, Merck, Darmstadt, Germany) were used to acidify and dilute all standards and samples.

### 2.6. Statistical Analysis

Statistical analysis of biosorption data was performed through a Student’s t-test to compare two groups of data *at p* < 0.05. All the analyses were performed using Software R version 3.5.1.

## 3. Results

### 3.1. Morphological Characterization of Electrospun and Electrosprayed Microstructures

#### 3.1.1. Characterization of Electrospun Fibers with and without Immobilized *L. sphaericus* CBAM5

**Figure 2:**
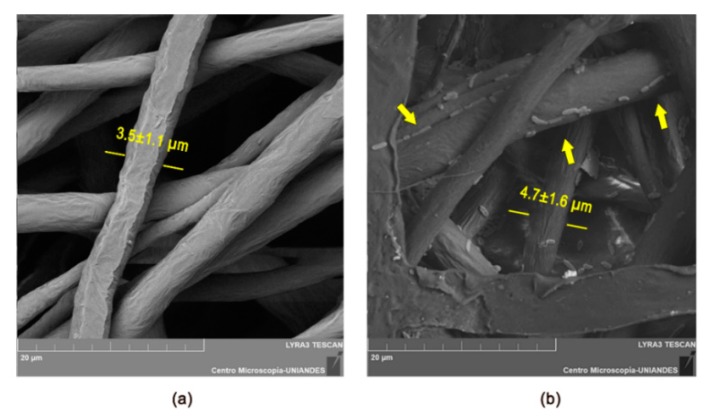
(**a**) Scanning electron microscopy (SEM) micrograph of a PCL membrane freshly synthesized (average diameter is 3.5 ± 1.1 µm). (**b**) SEM micrograph of a PCL membrane with immobilized bacteria (average diameter is 4.72 ± 1.64 µm).

The sub-micro-fibrous PCL mat was characterized by SEM analysis and its average diameter length and thickness were calculated. The average diameter obtained for the freshly synthesized PCL fibers was 3.5 ± 1.1 µm (Figure 2a) and this small value indicates that the mat has a large superficial area. Also, the fibers were randomly oriented over the collector and there was no evidence of pores formed in the mat.

The above characteristics of the PCL mat make it a suitable material for bacterial immobilization. This was proven by the determination of bacterial concentration immobilized using the detachment protocol. After the sonication of the PCL mats with bacteria, the concentration of *L. sphaericus* CBAM5 in saline solution was 5.59 × 10^7^ CFU/mL for the first cycle and more than 1 × 10^6^ CFU/mL after the second cycle. No significant differences were determined for the average diameter of the fibers after the bacterial immobilization. The final diameter was 4.72 ± 1.64 µm (Figure 2b). This shows that the inclusion of the mat in an aqueous medium does not affect its morphology. In addition, the SEM analysis showed the presence of bacteria attached to the PCL fibers as shown in Figure 2b. Finally, the average thickness of the mat after 3 h of PCL solution deposition by electrospinning was 66.34 ± 16.08 µm. 

#### 3.1.2. Characterization of Electrosprayed Microcapsules with *L. sphaericus* CBAM5 

**Figure 3:**
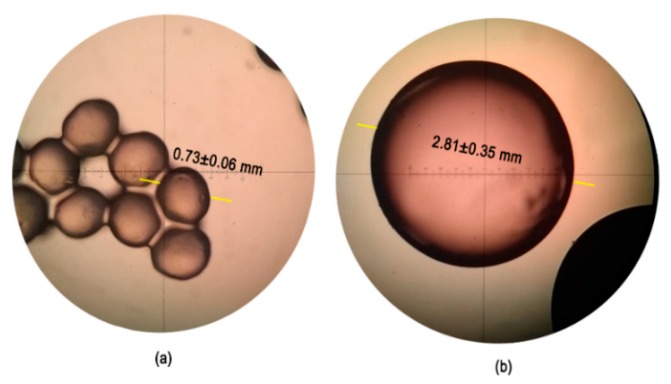
(**a**) Optical microscopy of the alginate microcapsules with *L. sphaericus* CBAM5 (average diameter is 0.73 ± 0.06 mm). (**b**) Optical microscopy of the dripped alginate capsules with *L. sphaericus* CBAM5 (average diameter is 2.81 ± 0.35 mm) [17].

The microcapsules obtained were observed using an optical microscope to measure the average diameter of the spheres. Figure 3 shows the measured diameter lengths and the average is 0.73 ± 0.06 mm, which is almost four times smaller than the diameter of capsules obtained by dripping the bacterial suspension with alginate over CaCl_2_ (data previously published, [17]). Moreover, the bacterial concentration in the microbeads was 1.17 × 10^6^ CFU/mL, demonstrating that no cell viability was lost after the exposure to high voltages during the electrospinning process. 

### 3.2. Gold Biosorption Assays with Electrospun and Electrosprayed Microstructures

#### 3.2.1. Biosorption of Gold by Inclusion of Micro-Fibrous Membranes with Immobilized *L. sphaericus* CBAM5

**Figure 4:**
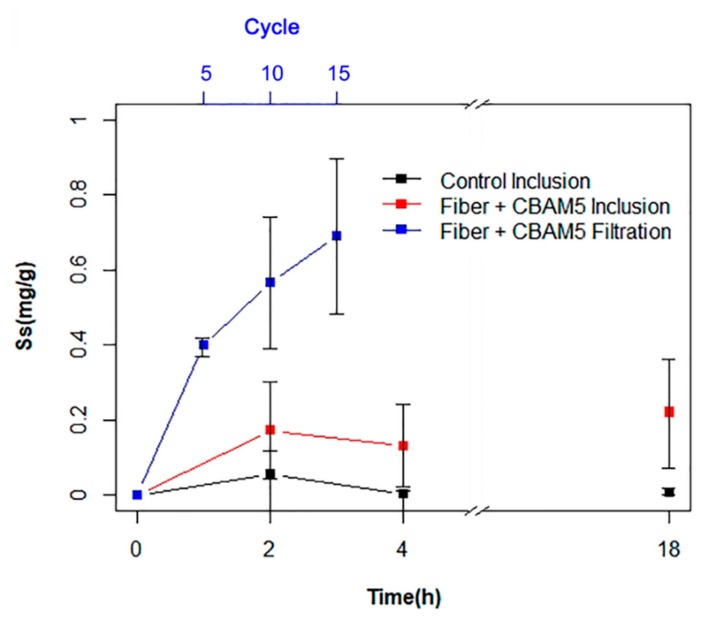
Gold (10 ppm) biosorption assays with *L. sphaericus* CBAM5 immobilized in PCL fibrous mats (Fiber + CBAM5) and the fiber without cells (Control) by inclusion in synthetic water and using a filtration system.

In order to assess the gold biosorption efficiency of *L. sphaericus* CBAM5 immobilized on a PCL membrane, FAAS was used to determine the gold concentration of the supernatant after the inclusion of the bio-composite mat in synthetic water with an initial concentration of 10 mg/mL HAuCl_4_ × 3H_2_O. As shown in Figure 4, the sorption capacity of the bio-composite electrospun mat was low and only 16% of gold was captured from the aqueous medium after 4 h of the bioassay and no gold removal was identified for the control assay. Immobilized *L. sphaericus* CBAM5 required a period of 120 h to remove 93% of the gold in the aqueous medium, while the neat PCL mat was able to remove 57% of the metal after that period of time. The difference between the biosorption efficiency of the bio-composite mat indicated that the presence of bacterial cells was responsible for the greatest proportion of gold capture. 

**Figure 5:**
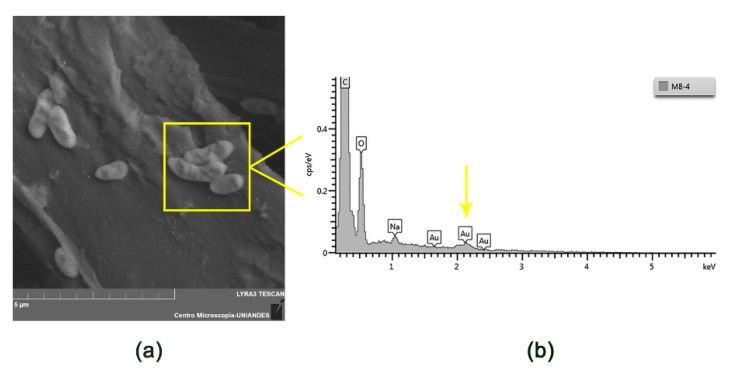
SEM micrographs of *L. sphaericus* CBAM5 cells (**a**) attached to a PCL microfiber seen with retro-dispersed electrons. (**b**) EDS analysis of image (**a**) showing the presence of Au in the cells.

The removal of gold by the attached cells of *L. sphaericus* CBAM5 was also confirmed using SEM analysis. Figure 5 shows that the bacteria have a bacilli morphology typical of *L. sphaericus* CBAM5 and the EDS analysis showed that the biomass was able to accumulate gold. 

#### 3.2.2. Gold Biosorption Performance of Micro-Fibrous Membranes with Immobilized *L. sphaericus* CBAM5 in a Filtration System

A filtration system was set up and several cycles of filtration were performed as an attempt to improve the gold biosorption efficiency of the micro-fibrous PCL mat with *L. sphaericus* CBAM5. Figure 4 shows that the PCL mat with immobilized bacteria had a high sorption capacity and removed 51% of the gold from the liquid sample after 15 cycles of filtration, which took a total time of 3 h. It is believed that greater efficiency could be achieved by performing more cycles. 

It can thus be suggested that placing the PCL mat in a continuous filtration system can significantly improve the removal of gold from liquid media when compared to a process by which the membrane is submerged in the synthetic water sample. 

#### 3.2.3. Biosorption of Gold by Microencapsulated Cells of *L. sphaericus* CBAM5 in Alginate

**Figure 6:**
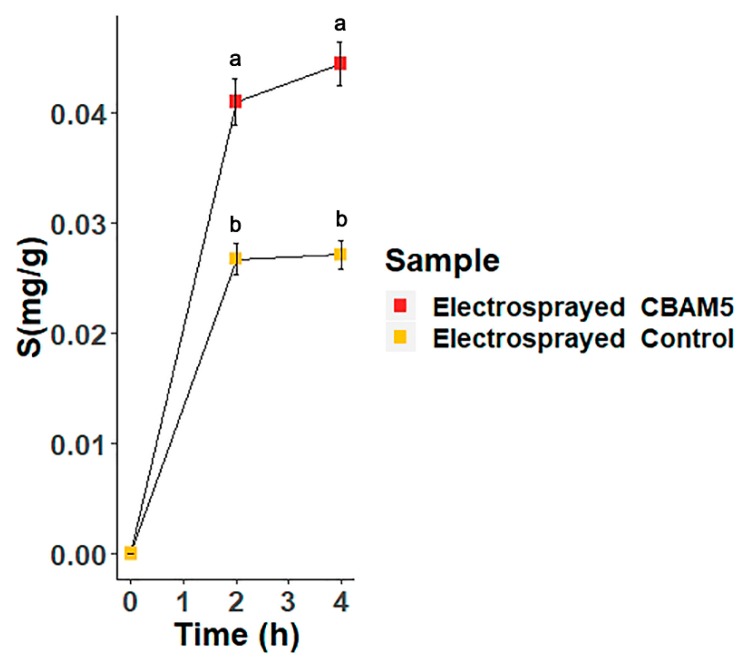
Gold Biosorption assay with *L. sphaericus* CBAM5 encapsulated in microcapsules of alginate (Electrosprayed CBAM5) and with microcapsules free of bacteria (Electrosprayed Control) for 4 h. Points at 2 and 4 h denoted by a different letter differ significantly at *p* < 0.05 according to a Student’s t-test analysis.

The efficiency of gold biosorption using the electrosprayed encapsulated bacteria in alginate was compared with the microcapsules free of *L. sphaericus* CBAM5. The results are presented in Figure 6. It was determined that the presence of bacteria in the matrix enhanced the capture of gold from the aqueous media. Moreover, the mg of gold captured were corrected using the total amount of alginate beads used in the bioassays in order to compare this with data previously published where alginate capsules were obtained by dripping the bacterial suspension into the CaCl_2_ solution [17]. By using dripped alginate capsules with bacteria, an efficiency of 0.02 mg of captured Au/g wet material was achieved after 2 h of the bioassay. While with electrosprayed microcapsules, an efficiency of 0.04 mg of captured Au/g wet material was obtained. It can be said that the electrosprayed alginate beads showed a more rapid capture of gold than the dripped alginate beads with *L. sphaericus* CBAM5. Also, after 4 h, the electrosprayed matrix captured almost doubled the amount of gold from the aqueous medium compared to the dripped alginate beads (data not shown).

## 4. Discussion

Several techniques have been proposed for the removal and recycling of metal ions from water bodies. Nevertheless, the use of sorption procedures with micro-structured materials is a suitable alternative to capture metal ions due to its high efficiencies, simple operation, and the flexibility of the materials used [2].

As such, two types of bio-composite micromaterials were developed in this study to enhance the gold biosorption efficiency of *L. sphaericus* CBAM5, a bacterium capable of binding with several metal ions such as Hg(II) [15] and Au(III) [16,17].

Firstly, PCL micro-fibrous mats were synthesized by using the electrospinning technique, which allowed the production of a sub-micro-scale structure with an average diameter of 3.5 µm, suitable for immobilization by adhesion of *L. sphaericus* CBAM5, as shown in Figure 2. Bacteria were immobilized by attachment to the randomly oriented fibers and due to their large superficial area [11]. An adsorption mechanism involving weak interactions like hydrogen bonds, hydrophobic bonds, or van der Waals interactions between the cells and the polymeric matrix occurred [20]. These weak interactions could lead to the release of bacteria from the membrane and these should be studied in the future.

After validating the high bacterial titers attached to the mats, an assay was performed to determine the gold biosorption efficiency of the bio-composites. The results of this study indicated that the capture of gold was a slow process (Figure 4) that required 120 h to remove 93% of the metal in solution. A possible explanation for this result may be the moderate wettability of the PCL polymeric structure due to its high hydrophobicity which restrains the water flux and thus, the contact of the solution with the bacteria immobilized [21].

Thus, a filtration system using the PCL mat was proposed in order to improve the efficiency of gold capture. Such a process allows a higher contact time of the solution with the membrane, and thus enables the immobilized bacteria and the PCL microstructure to interact and to efficiently adsorb gold ions in solution. In order to make a direct comparison between the efficiency of the two systems, the value of gold sorbed was corrected using the amount of PCL mats employed (g sorbed Au/g PCL). By doing this, a value of 0.56 g sorbed Au/(g PCL) was determined for the membrane after 10 cycles of filtration and 0.17 g sorbed Au/(g PCL) was found for the membrane submerged in the liquid sample for 2 h. The efficiency of the filtration system is therefore approximately three-fold greater than that of the inclusion system.

Li et al. reported a gold adsorption capacity of approximately 2.5 mmol/g using thioamide-group chelating nanofibers of polyacrylonitrile after about 12 h of exposure to a gold solution [22]. This value is comparable to the sorption capacity obtained in this work (2.8 mmol/g) by using a filtration system with the PCL bio-composite mat. However, it is worth noting that the time required in this study was only 2 h and the process to obtain the material did not require high temperatures and it consumed less resources.

PCL micro-fibrous mats’ high level of efficiency to capture gold may be explained by the fact that the morphology of the bio-composite brings several advantages during the sorption of gold from the aqueous medium, allowing it to capture the metal with or without immobilization with bacteria (Figure 4 and Figure 5). Its sub-micro-structure offers a large surface-to-volume ratio that provides several active sites for bacterial adsorption or metal binding and this reduces the amounts of material required for the assays. Also, the porosity of the material and its interconnectivity allows the water to permeate through filtration systems applied for water treatment [5]. The microfibers with immobilized bacteria have higher selectivity and therefore restrict the flow of specific substances or elements such as gold ions and allow the flux of water.

*L. sphaericus* CBAM5 is responsible for the great efficiency as these living bacteria present active and passive mechanisms for gold removal, such as absorption and adsorption, respectively [14]. In the case of adsorption, it has previously been shown that this bacterium presents an S-Layer protein that assembles in the cell wall and acts as an ion trap [18]. Moreover, it is known that this protein possesses functional groups with affinity to metals like Au(III) and thus, complexation may occur after the exposure of *L. sphaericus* CBAM5 to that metal ion. This can be observed in Figure 5, where a SEM analysis demonstrated the presence of gold in the surface of the bacteria. In addition, metabolically active cells of *L. sphaericus* CBAM5 are able to transport metal ions through efflux pumps or ion channels [23] and accumulate gold inside the cell [16].

The other approach to enhance the gold biosorption of *L. sphaericus* CBAM5 involved the microencapsulation of the living cells in an alginate matrix using electrospraying. By using a high voltage during the injection of the bacterial suspension with alginate into a CaCl_2_ solution, a large decrease in the capsules’ diameter was achieved and the resulting spheres were four times smaller than the ones obtained by dripping the alginate solution with no voltage supplied (Figure 3). Several properties of the injected solution, such as its conductivity due to the presence of ionic salts, favored the initiation of jetting and the formation of the Taylor cone, which is responsible for the decrease in the capsules diameter [24].

Notably, a high bacterial titer was determined in the microcapsules which shows that there was no considerable loss of cell viability after the exposure of the bacterial suspension to high voltage. These results demonstrated that the encapsulation in an alginate matrix acts as a protection layer for bacteria and it could also prevent their deterioration by factors such as low pH, UV light, oxygen, drought, and other stress conditions in the environment [25].

The gold biosorption efficiency of the microcapsules with *L. sphaericus* CBAM5 was determined and compared with the performance of the capsules with a millimeter scale diameter. The microcapsules showed a more rapid sorption of gold from the aqueous medium. The observed increase in the biosorption efficiency by microcapsules could be attributed to their small size and large surface-to-volume ratio [24], which increase the effective retention time of the solution and its exposure to bacterial cells. As seen in Figure 6, the sorption of gold could be divided into two processes. First, a rapid step that involves the absorption of the solution by the porous structure of alginate and the ad/absorption of gold by the encapsulated bacteria, and second, a process that reaches equilibrium with the saturation of the bio-composite [26]. Nevertheless, this should be verified in future work through a kinetic study and by analyzing more aliquots at different points in time.

In order to make a comparison between the two polymers employed in this study, Equation (2) was used to determine the sorption capacity of the microcapsules but instead of using the wet weight of the material, the dry weight of the alginate microbeads with *L. sphaericus* CBAM5 was employed as this value is comparable to the sorption capacity (Ss) of the PCL sub-micro-fibrous mat with immobilized bacteria. As shown in Table 1, the PCL mat placed in a filtration system yielded a higher gold capture efficiency than the electrosprayed alginate microcapsules with *L. sphaericus* CBAM5. This could be attributed to the higher surface area per gram of polymer obtained for the PCL mat due to the smaller size of the fibers compared to the diameter of the alginate microcapsules. This higher surface area allows a better distribution of the bacteria in the material and their effective interaction with gold. Moreover, the exposure of the PCL mat to the gold solution for ten cycles allowed a better contact time of the bacteria to the metal ions, improving their sorption capacity compared to the efficiency obtained using a batch system with the alginate microcapsules.

In conclusion, both micro-structured matrices were suitable for the immobilization and protection of *L. sphaericus* CBAM5, and they showed improved gold sorption performances so they could be applied to the recycling of this precious metal from industrial wastewaters, specially the PCL sub-micro-fibrous mats placed in a filtration system, as this showed the higher efficiency. Further research should be conducted to investigate the selectivity of both bio-composite materials by exposing them to a complex solution of metal ions and quantifying the sorption capacity of the micro-fibrous mats and microcapsules to each metal. Moreover, the recovery of gold from both micro-structured matrices should be studied using different desorption agents, such as thiourea. More efforts should be made to develop a scalable process in order to obtain larger amounts of these types of micro-structured-bio-composite materials.

## Figures and Tables

**Table 1:** Comparison between the gold sorption efficiency of the PCL bio-composite mat and the alginate microcapsules with *Lysinibacillus sphaericus* CBAM5, after 2 h of the bioassays.

Material	Size	Captured Au/(Weight Dry Material)
	µm	mg/g
PCL sub-micro-fibrous mat (Inclusion)	2.91 ± 0.36	0.17 ± 0.13
PCL sub-micro-fibrous mat (Filtration)	2.91 ± 0.36	0.56 ± 0.17
Electrosprayed microcapsules	730 ± 60	0.26 ± 0.01
